# Can we use lower extremity joint moments predicted by the artificial intelligence model during walking in patients with cerebral palsy in the clinical gait analysis?

**DOI:** 10.1371/journal.pone.0320793

**Published:** 2025-04-01

**Authors:** Firooz Salami, Mustafa Erkam Ozates, Yunus Ziya Arslan, Sebastian Immanuel Wolf

**Affiliations:** 1 Clinic for Orthopedics and Trauma Surgery, Heidelberg University Hospital, Heidelberg, Germany; 2 Department of Electrical Electronics Engineering, Faculty of Engineering, Turkish-German University, Istanbul, Turkey; 3 Department of Robotics and Intelligent Systems, Institute of Graduate Studies in Science and Engineering, Turkish-German University, Istanbul, Turkey; Ningbo University, CHINA

## Abstract

Several studies have highlighted the advantages of employing artificial intelligence (AI) models in gait analysis. However, the credibility and practicality of integrating these models into clinical gait routines remain uncertain. This study critically evaluates an AI model’s ability to predict lower extremity joint moments during gait in patients with cerebral palsy (CP). We employed a three-step approach to assess the feasibility of a previously developed AI model that predicted joint moments during walking for 622 patients with CP, using joint kinematics as input. First, we established clinically relevant thresholds for lower extremity joint moments, categorizing into three labels: acceptable (Green), acceptable with caution (Yellow), and unacceptable (Red). This categorization was based on the normalized root mean square error (nRMSE) between lab-measured and predicted joint moments. We explored the relationship between gait kinematics and joint moments by correlating the kinematic inputs with their respective output labels. Finally, we developed a linear discrimination analysis (LDA) model to predict labels for newly predicted joint. Assessing the validity of thresholds, an ANOVA one-way analysis and Bonferroni post-hoc statistical tests were performed to find significant differences between the nRMSE values for each label. The hip joint exhibited the largest population of Green labels (84%), while the ankle joint had the smallest (50%). Regressive differences in joint kinematics and gait profile scores were observed across all labels. The LDA model achieved an accuracy of 85.2% and an F-score of 92% for predicting Green label in hip joint moment. Additionally, more severe patient conditions were associated with an increase in Red-labeled predictions. Our findings highlight significant differences in nRMSE among labels, demonstrating the effectiveness of the proposed thresholds for labeling joint moments. Overall, the AI model’s performance was rated as moderate, and the three-step approach proved valuable for assessing the feasibility of AI models in clinical settings.

## Introduction

The use of artificial intelligence (AI) has grown significantly across various scientific fields, and motion analysis has not been left untouched in this regard [1,2]. AI models have provided the capability to look deeper into the musculoskeletal system and movement disorders, which were not achievable by conventional models [3–5]. Shin et al. discussed the benefits of AI in facilitating and increasing the efficiency of musculoskeletal imaging workflow in clinical applications in a review study [6]. In another study, Sharma et al. trained different AI models to estimate the kinematics and kinetics features obtained from conventional musculoskeletal models. They used inertial motion capturing data of five typically developed adults as inputs to the AI models and suggested the AI model as a promising approach [7].

Regarding In the field of motion analysis, AI models can be time- and cost-efficient by avoiding direct measurement of certain parameters through motion pattern recognition for diagnosis, classification, and prediction [8,9]. However, training an AI model necessitates a vast dataset, which limits the availability of studies focusing on patients with musculoskeletal disorders. Ozates et al. addressed this gap by training a convolutional neural network model to predict lower extremity joint moments during walking. They utilized retrospective gait data from 132 typically developed individuals and 622 patients with bilateral cerebral palsy (CP), drawing from a sizable database for their study [10].

In a more recent work, Xu et al. applied a metaheuristic optimization algorithm on 800 group gait datasets of typically developed subjects to determine the optimal gait feature, followed by the use of four classification algorithms, including artificial neural network models, to identify the selected gait features. They showed the practicality of using AI methods in clinical and sport applications [11]. In another study, they implemented a deep neural network model on running gait datasets and highlighted the critical role of the stance phase in gait recognition by AI models [12].

Despite these advancements, the most AI models in movement analysis remain within the research domain, and their performance is typically evaluated using standard metrics such as root mean square error (RMSE) and Pearson correlation [13]. While such criteria provide quantitative assessments to the AI model, they do not fully capture the clinical applicability of these models. A key unresolved question is whether AI models can be effectively employed in clinical settings, particularly for clinical gait analysis. To address this question, a more comprehensive evaluation of AI outputs from a clinical perspective is needed, involving patient-specific conditions during assessment. This broader approach will bring us closer to integrating AI into routine clinical practice.

**Fig 1:**
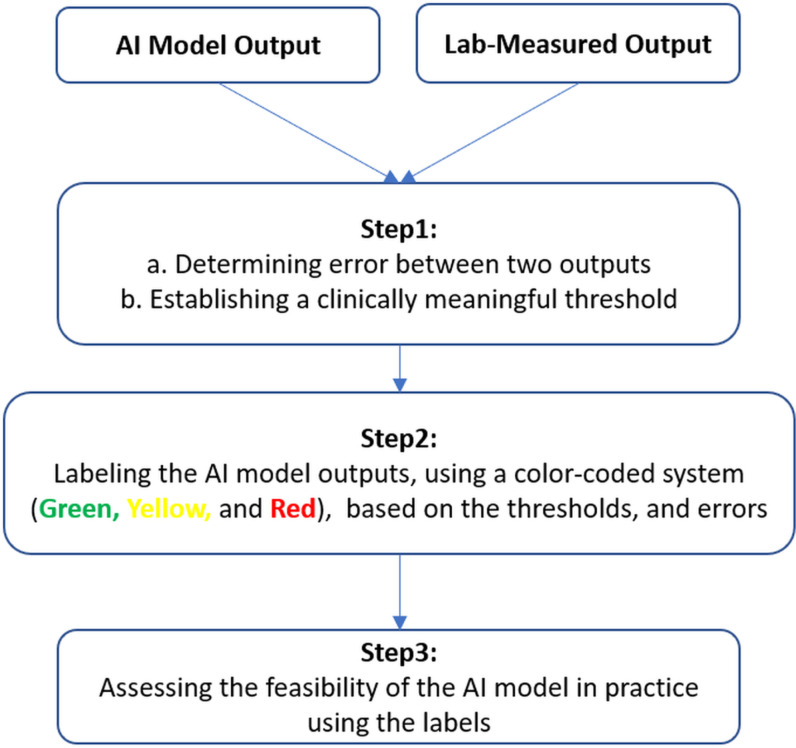
The schematic view of the three-step process approach.

In this follow-up study, we aimed to critically examine the AI model outputs from Ozates et al.‘s study [10] on patients with CP during walking. First, we defined clinically meaningful thresholds for lower extremity joint moments during walking to assess measurement errors. To the best of our knowledge, such thresholds have not been fully established yet. Next, we labeled the model outputs based on the thresholds, using a color-coded system (Green: acceptable, Yellow: acceptable with caution, and Red: unacceptable). Subsequently, we traced back the kinematic inputs and classified them according to their corresponding output’s label to evaluate the performance of the AI model in more detail. Finally, we assessed the potential use of the AI model in clinical gait routine (Fig 1).

## Method

### Data

This study utilized the same input and output data as Ozates et al. [10]. The dataset comprises anonymized retrospective gait kinematics and kinetics data from 622 CP patients with spastic diplegia, walking at self-selected speeds. The data analyzed in this retrospective study were part of a larger database established at the local University Clinics in the years 2000-2020. Only personnel that had regular legal access to the medical records retrieved patient data. They collected data in the time June and July 2022, and anonymized it in the same year August 1^st^. After this step, individual participants could not be identified anymore. The study was approved by the local ethical committee of the Heidelberg University Hospital (S-227/2021). Also, informed written consent was obtained from all subjects and/or their legal guardian(s).

All patients demonstrated the ability to walk barefoot without requiring any walking assistance. In the referenced study, a convolutional neural network model was trained to predict dorsi-plantar flexion, knee flexion-extension, and hip flexion-extension moments using laboratory-measured equivalent joint moments as targets and trunk, pelvis, hip, knee, and ankle kinematics as inputs. All participants were equipped with reflective skin-based markers in a setup according to the Plug-In Gait (PiG) protocol [14]. 3D motion data was captured with a twelve camera Vicon Nexus system (Vicon, Oxford Metrics, UK) and three force plates (Kistler Instruments, Winterthur, Switzerland).

The analysis focused solely on the stance phase of gait. The stance phase, defined as the interval between heel strike and toe-off for the left and right sides, was identified for each patient by an experienced operator in the gait laboratory and subsequently time-normalized to 60 points for consistency across all patients.

For more detailed information on the dataset and model, please refer to Ozates et al. [10].

### AI model (convolutional neural network)

Although the AI model used in this study was thoroughly detailed in Ozates et al. [10], a brief overview is provided here. The implemented machine learning architecture was based on a one-dimensional convolutional neural network, consisting of five convolutional layers with an increasing number of filters [128, 128, 512, 1024, 2048] and decreasing one-dimensional kernel sizes [3,5,10,15,30]. This architecture was intended to capture features for varying time intervals.

Following the convolutional layers, the model included ten densely connected layers, with a descending number of neurons [10000, 8000, 6000, 4000, 3000, 2000, 1000, 500, 250, 100], as a general approach for transforming information to the desired output size. The model was trained with a 10-fold cross validation algorithm to optimize the accuracy of the joint moment predictions [10]. Also, the model was trained separately for each joint.

### Joint moment threshold

A 5-degree measurement error, as the maximum allowable limit for gait kinematics, is well established in clinical routines [15]. However, no similar criterion exists for joint moments. In this regard, Meldrum et al. provided detailed results, including the standard error of measurement (SEM), along with the averaged peak of moments (Nm/kg) for the test-retest reliability of three-dimensional gait analysis for a cohort of 30 typically developed adults [16]. According to their results, joint moment SEM varied between 17% to 25% of the corresponded peak joint moment.

Lobet et al. reported the same parameters as Meldrum et al. for the 3D gait analysis of a cohort of patients with hemophilia experiencing blood-induced joint issues [17]. Same as previous study, their reported joint moment SEM varied between 12.1% and 24% of the maximal joint moment.

Further studies evaluated the relationship between peak joint moments and measurement errors, as well as clinically relevant changes in joint moments. Foucher (2016) showed that the average minimum clinically important improvement for the peak hip adduction moment after total hip arthroplasty for a cohort of females, with an average age of 61 years, is 0.87% Body Weight ×  Body Height (approximately equal to 14.75% of Nm/kg, assuming an average height of 1.73m) [18]. In another study, Miyazaki et al. [19] showed that knee osteoarthrosis increases 6.46 times with a 1% Body Weight ×  Body Height increase in the knee adduction peak moment (approximately 17% of Nm/kg). They found these values are useful in predicting knee osteoarthrosis after surgery. Additionally, Schwarze et al. [20] compared the effects of laterally wedged insoles and ankle-foot orthosis in patients with medial knee osteoarthritis. They concluded that a 5%-10% reduction in the maximum knee adduction moment during walking, typically achieved with lateral wedge insoles, may be clinically meaningful.

These studies demonstrated the significant variability in normalized peak joint moments, emphasizing the sensitivity of joint moments to prediction errors. To evaluate the prediction error of lower extremity joint moments in a clinically relevant context, we introduced two thresholds: the lower kinetic limit (LKL) and the upper kinetic limit (UKL).


$$LKL=10\%MaxMom^{*}$$ (1)

$$UKL=20\%MaxMom^{*}$$ (2)

where $MaxMom^{*}$ is the standardized peak joint moment.


$$MaxMom^{*}=\frac{\left|MaxMom–MeanMom\right|}{StdMom}$$ (3)

Applying these limits, it was possible to classify the joint moments into three labels using a color-coded system. The values smaller than LKL were considered acceptable (Green), those between LKL and UKL were considered acceptable but with caution (Yellow), and finally, values bigger than UKL considered unacceptable (Red).

### Kinematic features

Originally, the AI model predicted the joint moments using gait kinematics as input in all three planes. However, in this study, we limited our assessment to the sagittal plane during the stance phase.

Since the LKL and UKL are dimensionless criteria, the normalized RMSE (nRMSE) was used to address the difference between the measured and predicted joint moments of hip flexion/extension (hip moment, Nm/kg), knee flexion/extension (knee moment, Nm/kg), and ankle dorsiflexion/plantarflexion (ankle moment, Nm/kg) separately. The nRMSE was defined as the RMSE normalized to the peak-to-peak joint moment. It is widely recognized as a reliable metric for evaluating the outcome of the AI models. It offers a robust means of assessing the point-by-point magnitude difference between predicted and experimental joint moment time series [13,21,22].

For each patient, the lab-measured gait kinematics in the sagittal plane, including pelvic tilt (PelvicTilt), hip flexion/extension (HipFlx), knee flexion/extension (KneeFlx), and ankle dorsiflexion/plantarflexion (AnkleDorsi), were used for further evaluations. Additionally, the gait profile score (GPS) [23] was calculated for each patient, with the that the foot progression angle was excluded from the GPS calculation.

The hip, knee, and ankle joint moments for each patient were labeled using the LKL and UKL based on the nRMSE values (Table 1). The corresponding gait kinematics for each patient were then grouped according to these moment labels. Kinematic features, including maximal (MAX), range of motion (ROM), and mean (MEAN) values, were extracted for each label. GPS values were also calculated for each label. Additionally, to better understand the error distribution during the stance phase, it was divided into five subphases (coincide with initial contact, loading respond, mid stance, terminal stance and pre-swing). A boxplot was then generated for each joint moment during stance phase in each label, presenting the median as well as the first and third quartiles for the corresponding nRMSE (Fig 5).

**Table 1:** Population of each label classified using the LKL and UKL for the hip, knee, and ankle moments (Nm/kg) in number and percentage for the training group (500 subjects). The nRMSE mean ±  standard deviation of each label, along with their corresponding pvalues between labels, are also presented. The significant values are bolded (corrected p-value = 0.017).

	Hip Moment			Knee Moment			Ankle Moment		
	Population	%	nRMSE	Population	%	nRMSE	Population	%	nRMSE
Green	419	84	0.12 ± 0.03	297	60	0.14 ± 0.04	247	50	0.10 ± 0.03
Yellow	75	15	0.21 ± 0.04	166	33	0.23 ± 0.05	204	40	0.17 ± 0.04
Red	6	1	0.39 ± 0.15	37	7	0.40 ± 0.11	49	10	0.37 ± 0.25
p-value (Green vs. Yellow)			**<0.001**			**<0.001**			**<0.001**
p-value (Green vs. Red)			**<0.001**			**<0.001**			**<0.001**
p-value (Yellow vs. Red)			**<0.001**			**<0.001**			**<0.001**

**Fig 2:**
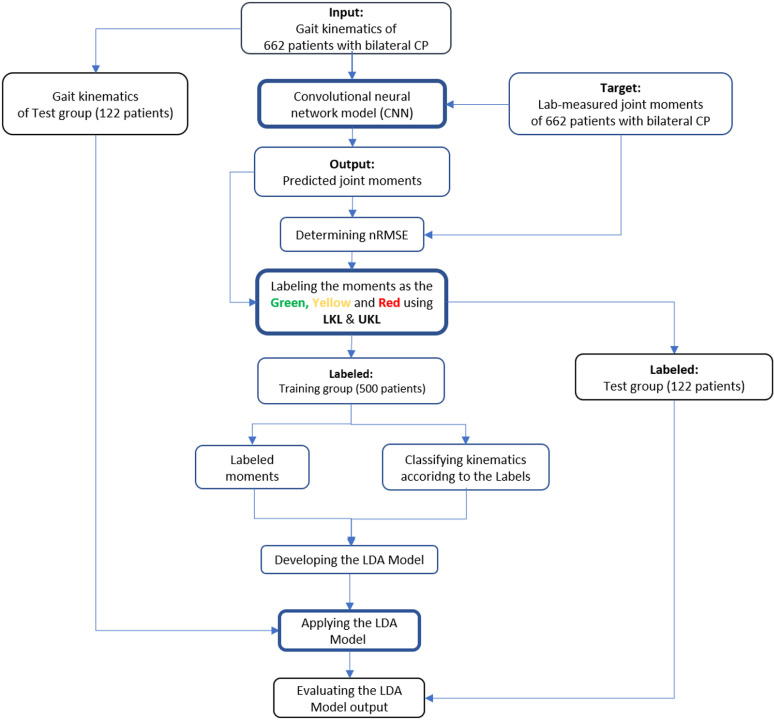
The data processing steps in details to illustrate the flow of model evaluation. Legend: LKL: lower kinetic limit, UKL: upper kinetic limit.

**Fig 3:**
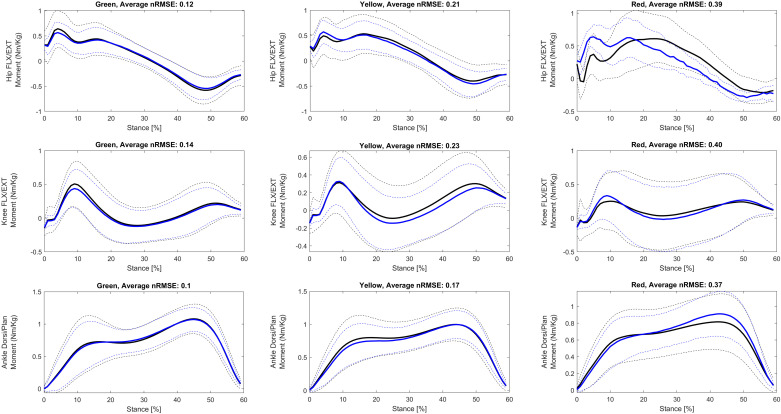
The training group nRMSE population distribution of three labels Green, Yellow, and Red for the hip, knee and ankle joint moments in the sagittal plane.

**Fig 4:**
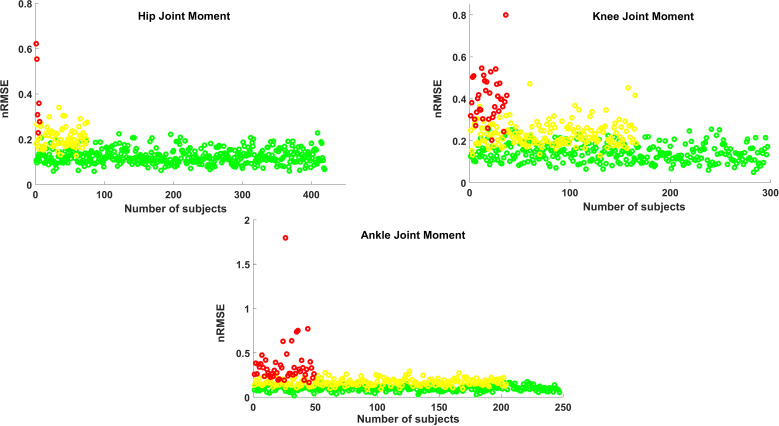
The averaged (solid line) and standard deviation (dashed line) hip, knee and ankle moments (Nm/kg) in the sagittal plane labeled using the LKL and UKL (Green, Yellow, and Red) for the training group during the stance (%). All data are time normalized to 60 points. Legend: The blue curves are the lab-measured moments and black curves are the moments predicted by the AI model.

**Fig 5:**
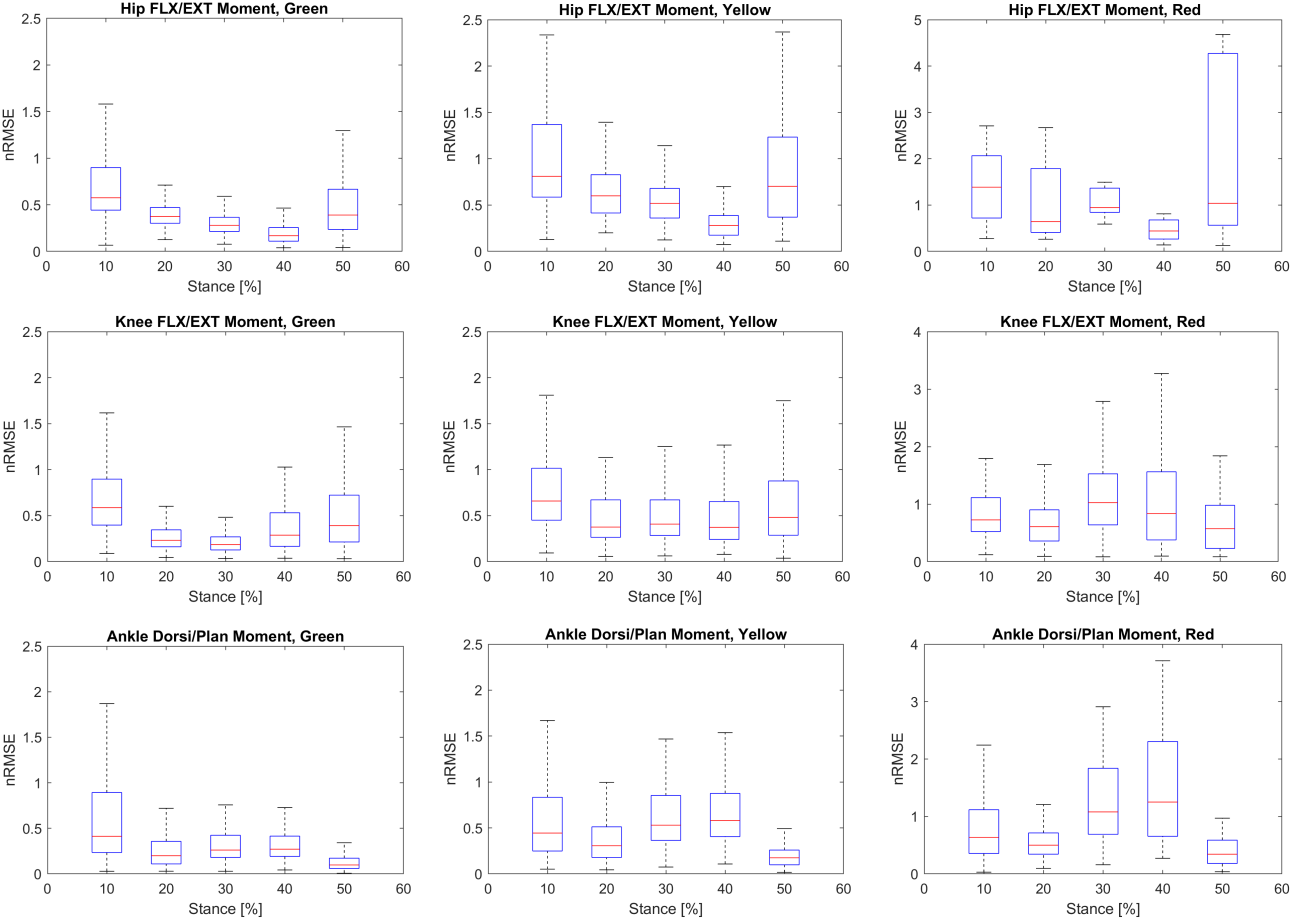
The boxplot of nRMSE distribution during stance for the hip, knee and ankle joint moments in each Green, Yellow and Red labels for the training group. Each plot presents median, first and third quartile ranges for five subphase of the stance.

### Feasibility assessment

The final step of the study focused on evaluating the potential of the AI model for use in clinical gait analysis. To this aim, the entire cohort was divided into a training group (500 subjects, 80%) and a test group (122 subjects, 20%). A Linear Discriminant Analysis (LDA) model [18] was then developed, using the gait kinematics and the corresponding moment labels (Green, Yellow, Red) from the training group as predictors and outputs, respectively.

Once trained, the LDA model was applied to the test group to predict the labels of the joint moments. This approach made it possible to assess how accurately the AI model’s predictions for new patients could be categorized into the correct label.

Two key metrics were used to evaluate the LDA model’s performance: model accuracy and F-Score [24]. Model accuracy measured the overall correctness of the LDA model in labeling the joint moments. The F-Score, which is the harmonic mean of precision and sensitivity, was applied to measure the model’s predictive performance for each label (Green, Yellow, Red) for each joint moment.

To assess the validity of the LKL and UKL thresholds, an ANOVA one-way analysis and Bonferroni post-hoc statistical tests were performed to find significant differences between the nRMSE values for each label (with a corrected p-value of 0.017). All calculations were done in MATLAB (© 1994-2024 The MathWorks, Inc, MA, USA). Fig 2 illustrates the entire data processing steps in details.

## Results

All the results presented in this section are specific to the sagittal plane (except for the GPS) during the stance phase for the training (Tables 1 and 2, and Figs 3–5) and test (Table 3) groups.

**Table 2:** Averaged (mean ±  std) kinematics features (MAX, ROM, MEAN, and GPS) during the stance corresponding to each label for each joint moment. Stance phase defined between foot strike and foot off for each left and right side and normalized to the 60 points.

	MAX (Deg)				ROM (Deg)				MEAN (Deg)				GPS (Deg)
**Hip Moment**	**Pelvic Tilt**	**Hip Flx/Ext**	**Knee Flx/Ext**	**Ankle Dorsi/Plan**	**Pelvic Tilt**	**Hip Flx/Ext**	**Knee Flx/Ext**	**Ankle Dorsi/Plan**	**Pelvic Tilt**	**Hip Flx/Ext**	**Knee Flx/Ext**	**Ankle Dorsi/Plan**	
**Green**	18.7 ± 6.6	40.6 ± 8.2	35.6 ± 10	12.7 ± 8.0	6.7 ± 3.1	40.5 ± 8.5	23.9 ± 7.4	21.7 ± 6.9	15.7 ± 6.3	17.8 ± 8.0	20.7 ± 11.7	5.8 ± 7.6	13.3 ± 3.5
**Orange**	19.5 ± 6.3	40.3 ± 9.4	39.5 ± 12.1	11.2 ± 9.6	7.8 ± 3.9	35.3 ± 10.4	22.6 ± 10.1	21.6 ± 6.2	15.9 ± 6.0	20.5 ± 9.6	25.2 ± 13.1	4.2 ± 9.2	14.7 ± 4.0
**Red**	16.3 ± 4.7	37.2 ± 7.4	40.3 ± 16.7	6.8 ± 10.3	7.9 ± 3.0	29.1 ± 9.0	19.1 ± 7.3	19.9 ± 5.4	12.9 ± 5.6	22.1 ± 10.8	28.7 ± 18.7	0.5 ± 9.7	16.4 ± 6.0
**Knee Moment**													
**Green**	18.8 ± 6.5	41.1 ± 7.4	35.8 ± 9.4	12.9 ± 7.6	6.6 ± 3.2	41.9 ± 8.5	24.5 ± 8.0	21.8 ± 7.1	15.8 ± 6.3	17.7 ± 7.2	20.7 ± 10.6	6.0 ± 7.2	13.2 ± 3.1
**Orange**	19.3 ± 6.6	40.0 ± 9.4	36.9 ± 11.4	12.4 ± 7.8	7.3 ± 3.4	36.2 ± 8.9	23.0 ± 7.6	21.3 ± 6.2	16.0 ± 6.2	19.3 ± 9.5	22.0 ± 13.2	5.5 ± 7.3	13.9 ± 4.1
**Red**	17 ± 6.9	38.2 ± 10.4	37.1 ± 15.9	8.6 ± 13.5	7.2 ± 3.1	36.0 ± 8.7	19.4 ± 6.8	22.5 ± 6.9	14.0 ± 6.5	18.6 ± 10.9	24.6 ± 17.7	2.0 ± 13.4	15.0 ± 5.2
**Ankle Moment**													
**Green**	19.3 ± 6.4	41.2 ± 8.2	36.1 ± 8.7	14.1 ± 6.3	6.6 ± 3.3	40.9 ± 9.2	24.6 ± 7.3	21.9 ± 6.6	16.3 ± 6.0	18.2 ± 7.7	20.7 ± 9.2	6.7 ± 5.8	13.1 ± 3.0
**Orange**	18.3 ± 6.8	40 ± 8.4	36.6 ± 12.1	11.5 ± 8.9	6.9 ± 3.2	39.1 ± 8.1	23.1 ± 7.9	21.6 ± 7.3	15.2 ± 6.4	18.1 ± 8.8	22.1 ± 13.9	5.1 ± 8.8	13.8 ± 4.2
**Red**	18.7 ± 6.6	39.2 ± 8.9	36.0 ± 13.4	7.7 ± 11.6	7.8 ± 3.6	35.3 ± 10.8	21.1 ± 9.6	20.7 ± 5.0	15.2 ± 6.7	19.4 ± 9.6	22.6 ± 16.5	1.62 ± 11.3	15.0 ± 4.3

**Table 3:** Population of Green, Yellow, and Red: classified using the LKL and UKL (LKL & UKL), predicted using the LDA model (LDA), and the matched between classified and predicted labels (Matched) for the test group (122 subjects). F-Score (%) and model accuracy (%) of the LDA model are also presented. The last raw presents the results for the sum of the Green and Yellow labels.

	Hip Moment					Knee Moment					Ankle Moment				
	**LKL & UKL**	**LDA**	**Matched**	**F-Score (%)**	**Model Accuracy (%)**	**LKL & UKL**	**LDA**	**Matched**	**F-Score (%)**	**Model Accuracy (%)**	**LKL & UKL**	**LDA**	**Matched**	**F-Score (%)**	**Model Accuracy (%)**
**Green**	107	119	104	92.0	85.2	69	98	53	60.9	44.2	69	88	49	61.2	49.2
**Yellow**	14	0	0	–		46	15	1	2.8		45	27	11	26.2	
**Red**	1	3	0	–		7	9	0	–		8	7	0	–	
**Green** + **Yellow**	121	119	118	98.3	96.7	115	113	106	92.9	86.8	114	115	107	93.4	87.7

Table 1 presents the distribution of each label (Green, Yellow, and Red) classified using the LKL and UKL for the hip, knee, and ankle moments (Nm/kg) in the training group (500 subjects). The averaged nRMSE for each label was also presented.

The largest and smallest populations for the Green label belonged to the hip (419, 84%), and ankle (247, 50%) moments, respectively. Conversely, the ankle moment had the largest populations for both Yellow (204, 40%) and Red (49, 10%), whereas the hip moment had the smallest populations for these labels (75, 15% for Yellow and 6, 1% for Red). Significant differences in nRMSE values were observed between all labels for all joints (p <  0.001).

Table 2 presents the averaged kinematic features (MAX, ROM, MEAN, and GPS) for PelvicTilt, HipFlx, KneeFlx, and AnkleDorsi angles (deg) across all labels. All kinematics features and GPS averaged values in all labels for all joint moments showed regressive trend from Green to Red, e.g., the averaged GPS values increased from Green to Red for the hip, knee, and ankle moments, with values of 13.3° to 16.4°, 13.2° to 15.0° and 13.1° to 15.0°, respectively.

Table 3 the LDA model results for the test group (122 subjects). The highest Green match between labeled (LKL & UKL) and predicted (LDA) moments was observed for the hip moment (104), while the ankle moment had the lowest (49). No Red matches were identified for any joint moments. The highest accuracy of the LDA model was for the hip moment (85.2%), whereas the accuracies for the knee and ankle moments were below 50% (44.2% and 49.2%, respectively). The F-Score was also highest for the hip moment (92.0%) and almost the same for the knee and ankle moments (60.9% and 61.2%, respectively). The model accuracy and F-Score for the Green+Yellow condition ranged from 86.8% to 96.7% and 92.9% to 98.3% for the hip and knee moments, respectively.

Fig 3 demonstrates the population distribution of nRMSE values across the Green, Yellow, and Red labels for the hip, knee, and ankle joint moments in the training group.

Fig 4 illustrates the hip, knee, and ankle moments (Nm/kg) in the sagittal plane during the stance phase, grouped by the LKL and UKL for the training group. The average nRMSE for each label was reported in the title of each plot, providing insight into the predictive accuracy of the joint moments.

Fig 5 shows the boxplot of nRMSE distribution, including the median, and the first and third quartile ranges, across the five subphases of stance for each joint moment and label for the training group. For the Green label, the first subphase exhibited the highest error in the hip, knee, and ankle joint moments. In the Red label, the highest error occurred during the final subphase for the hip moment, while the ankle and knee joint moments showed the maximum error in the fourth subphase.

## Discussion

In this study, we proposed a three-step approach (Fig 1, 2) to assess the outcome of an AI model developed to predict lower extremity joint moments during walking in patients with CP [10]. The aim was to evaluate the model’s practicality in clinical routine.

While conventional metrics such as RMSE and Pearson correlation primarily focus on evaluating the precision and accuracy of the AI model itself, these metrics alone do not offer a comprehensive assessment from a clinical perspective. In this study, we proposed clinically meaningful thresholds as a practical and intuitive metric for clinicians to evaluate the outcomes of the AI model. This approach is straightforward yet robust, providing an overarching perspective on the reliability of the AI-generated results. the color-coded system is more tangible and applicable for clinicians, aiding them in making more informed and effective clinical decisions.

The first step involved establishing clinically meaningful thresholds for lower extremity joint moments during walking, as no such thresholds have been fully defined. In the second step, we classified the joint moments into three labels (Green: acceptable, Yellow: acceptable with caution, and Red: unacceptable) based on the computed errors and established thresholds. Additionally, we grouped the AI model inputs (joint kinematics during walking in the sagittal plane) according to their corresponded joint moment labels, investigating how changes in kinematics correlates with the categorized labels. At the last step, we assessed the feasibility of the AI model. To do so, we developed an LDA model to detect the label of a newly joint moment predicted by the AI model. The joint moments were analyzed during the stance phase. The gait events were set manually because patients with CP show various event characteristics such as forefoot first contact and foot flat contact. Up to now there is not yet a method which is robust to detect these characteristics well enough, hence the manual process. It may be somewhat subjective and imprecise but less prone to more severe errors in timing.

Our findings showed significant differences in nRMSE (p <  0.001) among all labels (Table 1), demonstrating that our proposed thresholds (LKL and UKL) effectively classified joint moments in the presence of deformities (Figs 3, 4). The motivation behind introducing the LKL and UKL was to avoid relying solely on a rigorous method for evaluating and labeling the predicted joint moments, which lacks sufficient clinical and biomechanical relevance. The standardized joint moment definition accounts for both the distribution of joint moments throughout the stance phase and the peak joint load. Since, unlike kinematic errors, kinetic errors tend to exhibit a broader range of variation two 10% and 20% thresholds were proposed based on practical experience in routine gait data assessments and the range of errors reported in the literature. To the best of our knowledge, these thresholds are introduced for the first time in evaluating the validity of gait kinetics measures.

Technically, if the relative error between the predicted and measured moment (e.g., nRMSE) was below a specified threshold (LKL), we expected that the relative error would not (or only slightly) influence our interpretation of the predicted moment. The AI model demonstrated the best performance for the hip joint moment, with 84% labeled as Green (acceptable) and only 1% as Red (unacceptable). Conversely, for the ankle joint moment, with 10% labeled as Red and 50% as Green, the AI prediction was rather poor. As for the knee joint moment, results were slightly better than the ankle moment (60% Green, and 7% Red), suggesting moderate performance. Considering the average results for the entire training group population (65% Green, 29% Yellow, and 6% Red), the overall performance of the AI model was rated as moderate.

The results for the kinematic features presented in Table 2. Although the differences in sample sizes between labels for each joint moment (e.g., Green =  419, Yellow =  75, Red =  6) made it challenging to draw definitive conclusions, the average values of the features showed a progressive regression from Green to Red. These values showed an increasing severity of gait kinematics from Green to Red. For instance, the maximum knee flexion/extension increased from 35.6 ±  10° (Green) to 40.3 ±  16.7° (Red), suggesting a more severe crouched gait in patients with CP [25]. Along with gait kinematics features, the averaged GPS values increased from Green to Red (ranging from 13.3 to 16.4°, 13.2 to 15.0°, and 13.1 to 15.0°) for the hip, knee, and ankle moments, respectively. The GPS values indicated that kinematics had the tendency to deviate further from typically developed reference through Green to Red for all the joint moments. Overall, by comparing the kinematic results with the population of labels, it can be concluded that a higher severity of deformities and a more variable gait pattern correlate with an increased likelihood of the predicted joint moment being labeled as Red.

Fig 5 illustrates the error distribution during stance for each joint moment. The highest errors for all joints occurred during initial contact and loading response, particularly in the Green and Yellow labels. This may be attributed to the foot striking the ground and the associated shock absorption. The elevated error during terminal stance and pre-swing phases for the knee and ankle joints in the Red label could be related to the contralateral foot’s initial contact and preparing for foot off, leading to slight instability. However, drawing a definitive biomechanical conclusion for the hip in the Red label is challenging due to the limited number of subjects (6 subjects). Overall, all joints in the Green and Yellow labels exhibited relatively smooth and low errors during midstance, which could be due to reduced variability and the smoother progression of the moment curves.

The better prediction for the hip moment may be attributed to the smaller variations in the hip moment curves throughout the gait stance phase compared to the knee and ankle joints, enabling the AI model to adapt more effectively. Also, the Plug-in-Gait simplified foot segment, which lacks several important characteristics of the foot, may have contributed to the relatively poorer prediction outcomes for the ankle moment, particularly in subjects with foot deformities.

A linear discriminant model was developed to assess the potential of the AI model for clinical routine use. LDA provides a clear and interpretable decision boundary regarding pattern classification in not very large training datasets [26–28]. The model accuracy and F-Score presented in Table 3 were used to evaluate the LDA model’s effectiveness in predicting the correct labels and its strength, and to evaluate the strength of model in detecting the correct labels for test group, respectively. This approach allowed the study to assess the model’s ability to generalize to new data and its potential for integration into clinical gait analysis practices. The LDA model demonstrated promising performance for the hip moment, achieving an accuracy of 85.2% and an F-Score of 92.0% in the Green category, while its performance for the knee and ankle moments was average. Additionally, both the model accuracy and F-Score for all joint moments increased significantly when the two labels of Green and Yellow were combined. Building on the previously discussed results, the more successful the AI model is, the more accurate the detections of the LDA model will be. In this context, LDA performed well by enabling effective joint-to-joint comparisons. Although utilizing a classifier may result in the loss of certain gait characteristics in patients with CP, which could impact feasibility, applying thresholds and combining the CNN with LDA allowed us to effectively assess the feasibility of the AI model for clinical use.

Overall, for a new patient, if the AI model predicts the hip moment as Green (via the LDA model), the outcome can be considered acceptable with a high degree of confidence. However, predictions for the knee and ankle moments should be approached with caution. If labeled as Yellow or Red, it is recommended to categorize these as Red (unacceptable) for all joint moments.

Several factors may contribute to the AI model’s performance limitations, including the complexity of joint biomechanics (e.g., the relatively simple ball-and-socket hip joint compared to the more complex ankle joint), the high variability in gait patterns among patients with CP, the limited size of the training dataset (in relation to the pattern variations), and the architecture of the AI model. To address these challenges, we recommend enhancing model performance by employing more sophisticated machine learning methods, incorporating more accurate biomechanical models, and increasing the training dataset size by including a greater variety of biomechanical signals.

There are limitations to this study. Although the AI model utilized kinetics and kinematics in all three planes, this study focused exclusively on the sagittal plane moments and angles. This limitation may affect the accuracy of the LDA model, indicating a scope for improvement in the label predictor model. A thorough assessment of kinematic features in the frontal and transverse planes, combined with additional biomechanical data such as electromyography signals, will be crucial to gain a deeper understanding of the relationship between the AI model’s reliability and its biomechanical relevance. This would help provide a more comprehensive evaluation of the model’s performance across all planes of motion. The Plug-in-Gait model may overlook certain biomechanical and anatomical characteristics of the patient, which could introduce errors in the moment prediction, especially in cases with atypical gait patterns or deformities. Future work should focus on enhancing the AI model to achieve better predictions of joint moments in the presence of severe deformities. Additionally, further studies, including data from typically developed individuals, are necessary to have a better understand of the relationship between the proposed LKL and UKL and the presence of deformities. Further studies involving patients with varying conditions are necessary to generalize the three-step assessment framework to a broader clinical context.

In conclusion, the three-step assessment through the labeling the joint moments has proven to be a valuable approach that can be implemented to assess the practicality of the AI models for patients with CP. The three-color coded system is simple and practical in classifying the data. The thresholds introduced in this study were highly efficient in labeling and distinguishing the moments and can be used as standalone features for evaluating gait kinetics. Utilizing the AI model to predict the joint moments in the sagittal plane for patients with CP is recommended for assessing hip flexion/extension moment, particularly for patients with mild or moderate severity. However, the general performance of the AI model is still moderate. While AI models can be time- and cost-effective and facilitate clinical applications, further development is necessary before they can be considered adequate substitutes for daily clinical gait measurement routines.

## Supporting information

S1 Table The maximal, mean, and standard deviation values of the hip, knee and ankle joint moments for lab-measured and predicted (AI model) for each patient. Also, nRMSE between lab-measured and predicted moments and gait profile scores for each patient.
(XLSX)
